# Patient Self-Management of Oral Anticoagulation with Vitamin K Antagonists in Everyday Practice: Efficacy and Safety in a Nationwide Long-Term Prospective Cohort Study

**DOI:** 10.1371/journal.pone.0095761

**Published:** 2014-04-18

**Authors:** Michael Nagler, Lucas M. Bachmann, Pirmin Schmid, Pascale Raddatz Müller, Walter A. Wuillemin

**Affiliations:** 1 Division of Haematology and Central Haematology Laboratory, Luzerner Kantonsspital, Lucerne, and Department of Haematology and Central Haematology Laboratory, Inselspital University Hospital, Berne, Switzerland; 2 medignition Inc., Zug, Switzerland; 3 Division of Haematology and Central Haematology Laboratory, Luzerner Kantonsspital, Lucerne, Switzerland; 4 Division of Haematology and Central Haematology Laboratory, Luzerner Kantonsspital, Lucerne, Switzerland; 5 Division of Haematology and Central Haematology Laboratory, Luzerner Kantonsspital, 6000 Lucerne, and University of Berne, Berne, Switzerland; University of Glasgow, United Kingdom

## Abstract

Patient self-management (PSM) of oral anticoagulation is under discussion, because evidence from real-life settings is missing. Using data from a nationwide, prospective cohort study in Switzerland, we assessed overall long-term efficacy and safety of PSM and examined subgroups. Data of 1140 patients (5818.9 patient-years) were analysed and no patient were lost to follow-up. Median follow-up was 4.3 years (range 0.2–12.8 years). Median age at the time of training was 54.2 years (range 18.2–85.2) and 34.6% were women. All-cause mortality was 1.4 per 100 patient-years (95% CI 1.1–1.7) with a higher rate in patients with atrial fibrillation (2.5; 1.6–3.7; p<0.001), patients>50 years of age (2.0; 1.6–2.6; p<0.001), and men (1.6; 1.2–2.1; p = 0.036). The rate of thromboembolic events was 0.4 (0.2–0.6) and independent from indications, sex and age. Major bleeding were observed in 1.1 (0.9–1.5) per 100 patient-years. Efficacy was comparable to standard care and new oral anticoagulants in a network meta-analysis. PSM of properly trained patients is effective and safe in a long-term real-life setting and robust across clinical subgroups. Adoption in various clinical settings, including those with limited access to medical care or rural areas is warranted.

## Introduction

Patient self-management (PSM) has become a promising concept for various chronic illnesses such as diabetes, high blood pressure or chronic obstructive pulmonary disease [1]–[3]. For patients with arthritis and perhaps also for patients with asthma PSM has shown to improve outcomes and also reduce cost (summarised in Bodenheimer et al. 2002 [1]). The role of PSM in long-term anticoagulation therapy to prevent thromboembolic events has been vividly discussed recently [4]–[10]. Proponents claim that PSM should be seen as the new benchmark for other management schemes and anticoagulation therapies. They draw on several clinical trials and meta-analyses documenting better anticoagulation control, less thromboembolic complications, increased quality of life, and, in part, a reduced mortality if compared with usual care [4]–[6], [11]–[16] Some large scientific societies have adopted their view and recommend discussing PSM with eligible patients [17]–[20].

Opponents in return interpose that evidence on long-term safety and treatment control in clinical subgroups is sparse. Moreover, several authors recently questioned the generalizability of available trial evidence, because patients included in randomised-controlled studies are prone being heavily selected [4], [5], [7]–[10], [15], [18], [21], [22]. Discrepancies between data obtained in clinical trials and daily practice is regarded as a particular issue in anticoagulation therapy [21], [22]. A recent systematic review, identifying a relevant lack of evidence thus called for population-based cohort studies to clarify the long-term efficacy and safety in a real-life setting [4].

To contribute to the discussion, we performed a nationwide, prospective cohort study determining efficacy and safety of PSM in a long-term real-life setting and with view to salient clinical subgroups such atrial fibrillation, mechanical heart valves, venous thrombosis and in elderly patients. To contextualize the results of our cohort, we additionally performed a network meta-analysis of major thromboembolism trials to compare efficacy parameters with VKA standard care, rivaroxaban, dabigatran, and apixaban.

## Methods

### Study Population

In this prospective cohort study, all patients trained for PSM within the initiative “coagulationcare” in Switzerland between 1998 and 2009 were included. This nationwide initiative is maintained by the charitable foundation of the same name. It trains about 90% of all Swiss patients and 95% of patients in German-speaking Switzerland. Observation period was the time span between PSM training and 31^th^ of December 2010.

### Patient Selection

All patients that were referred for PSM training have been trained without applying any type of selection. Patients were referred by the family physician, a specialist, or hospital staff. Information on PSM training was provided by presentations at scientific meetings, articles of national journals, websites, and in particular by patient organisations. Although, theses information were prepared in accordance with existing guidelines [18], [19], [23], [24], systematic selection criteria were not provided.

### Ethics Statement

The study received Ethics approval by the local review board (Kantonale Ethikkommission Luzern; #422) and all participants provided written informed consent.

### PSM Training

With a view on international PSM practice and in accordance with published guidelines, a structured training programme was developed [18], [19], [23]–[25]. Details of the programme have been published previously [26]. In brief, patients had to attend a one-day training course at one of the study centres (Lucerne, Berne, Basel, Zurich, or Olten). A team of specialized physicians and paramedic staff taught all aspects of oral anticoagulation in several theoretical and practical sessions. In the theoretical part, participants learned about interactions with other drugs, interference with nutrition, the effects of concomitant illness on VKA treatment, the most common adverse events and safety measures when travelling. Moreover, instructions on the proper handling of the coagulation monitor were provided. Participants also learned how to interpret and document the results, how to use the dosing algorithm and adjustment dosages, and aspects of quality control. Practical training followed the lectures. After completion of the one-day course, participants entered the training phase for several weeks that included consultations with the family physician and parallel determinations of the international normalised ratio (INR) value.

Within this second phase, patients and family physicians were supported by online-material, e-mail contact and a 24-hour hotline. After completion of the training phase, all participants returned to the study centre for a one-hour check-up visit with a specialised physician. During the visit participants repeated the learning matters and performed an INR testing and dose adjustment under supervision. A typical training package is illustrated in Table S2.

Participants were advised to do INR testing at least every two weeks and to get parallel measurements with the family physician two times a year. INR measurements were performed using the portable coagulometer CoaguChek XS (CoaguChek S until 2005 and CoaguChek until 2000; Roche Diagnostics, Basel, Switzerland), showing adequate accuracy, also in the hands of patients [27], [28].

### Definition of Outcomes

The following events were defined as primary outcome parameters: (i) any death, (ii) venous and arterial thromboembolic events (TE) (including deep vein thrombosis, pulmonary embolism, myocardial infarction, ischemic stroke or transient ischemic attack, systemic embolic events, other thromboembolic events), and (iii) major bleeding (defined as lethal bleeding, clinical overt bleeding, bleeding in critical organs, bleeding that needs transfusions, and any bleeding that necessitates medical consultation with diagnostic or prophylactic interventions). Secondary outcome parameters were (iv) event-related death, (v) event-related death and death of unknown cause, (vi) composite of TE and unknown death, (vii) intracranial bleeding events, and (viii) time in therapeutic range (%TIR). Death was regarded as event-related in case of a thromboembolic event (including myocardial infarction) or bleeding event. Death was regarded as not event-related in case of infection, cancer or perioperative death.

### Data Acquisition

At the time of inclusion, the following data were recorded: age, sex, indication for oral anticoagulation, starting date of anticoagulation and data regarding health insurance. As long as first patients were included in 1998 and risk assessment regarding thromboembolic and bleeding complications were not done regularly at this time, the presence of heart failure, hypertension, diabetes, previous stroke or bleedings, renal failure, or vascular disease were not recorded.

For follow-up, data on TE, bleeding events or deaths were obtained by a standardized questionnaire. In case of deaths, complications or hospitalisations, medical records and additional information were requested by contact with the family physician, hospitals, the relatives and the authorities. Three separate investigators controlled data recording. Two scientists, who are physicians treating PSM patients regularly (M.N. and W.A.W.), discussed unclear cases. In absence of an agreement, classifications were made for the disadvantage of the treatment. Patients were requested and reminded to send their INR- and dosage-documentation to the study centre.

### Statistical Analysis

For determination of complications, only patients aged 18 or more were considered. We calculated the percentage of time in therapeutic range according using linear interpolation (Rosendaal method) [29], [30].

For each of the three outcome parameters (overall mortality, TE, and major bleedings) we performed a univariate survival analysis and estimated differences in survival by indications of oral anticoagulation (atrial fibrillation, venous thromboembolism and mechanical heart valve), age (<50 vs.≥50 years), or sex (male vs. female). Differences in survival were tested using the logrank test if appropriate. Analyses were performed using the Stata 11.2 statistics software package. (StataCorp. 2009. Stata Statistical Software: Release 11. College Station, TX: StataCorp LP).

### Network Meta-analysis

To set our results into context, we performed a network meta-analysis comparing efficacy of PSM with other important anticoagulation schemes. We selected six major studies, investigating the efficacy of Rivaroxaban 20 mg, Apixaban 10 mg, Warfarin or another VKA and Dabigatran 150 mg to reduce the occurrence of recurrent thromboembolism and enrolling 9,872 patients were used for the analysis [31]–[36]. Dabigatran 150 mg was chosen for comparison, because it has demonstrated a superior efficacy than 110 mg. Whenever possible we used results from intention to treat analysis. Data were abstracted into 2 by 2 tables. A network meta-analysis was performed applying a recently published method [37]. In brief, a logistic regression model was used. Drug and dosage, creating a unique code for each treatment, were entered as covariates. To preserve randomization within each trial, we included an indicator variate for each study. This variate adjusted for all differences in risk profiles and study setup between trials. The event outcome for each single treatment arm was used as the dependent variable. From this regression model we estimated the odds ratio (OR) and 95% confidence intervals between placebo and all other treatment options. To compare the efficacy parameter with the results from our PSM study, we summarized the frequency of occurrence of the outcome in the placebo arms and calculated the corresponding OR and confidence interval.

## Results

### Patients and Follow-up

**Figure 1:**
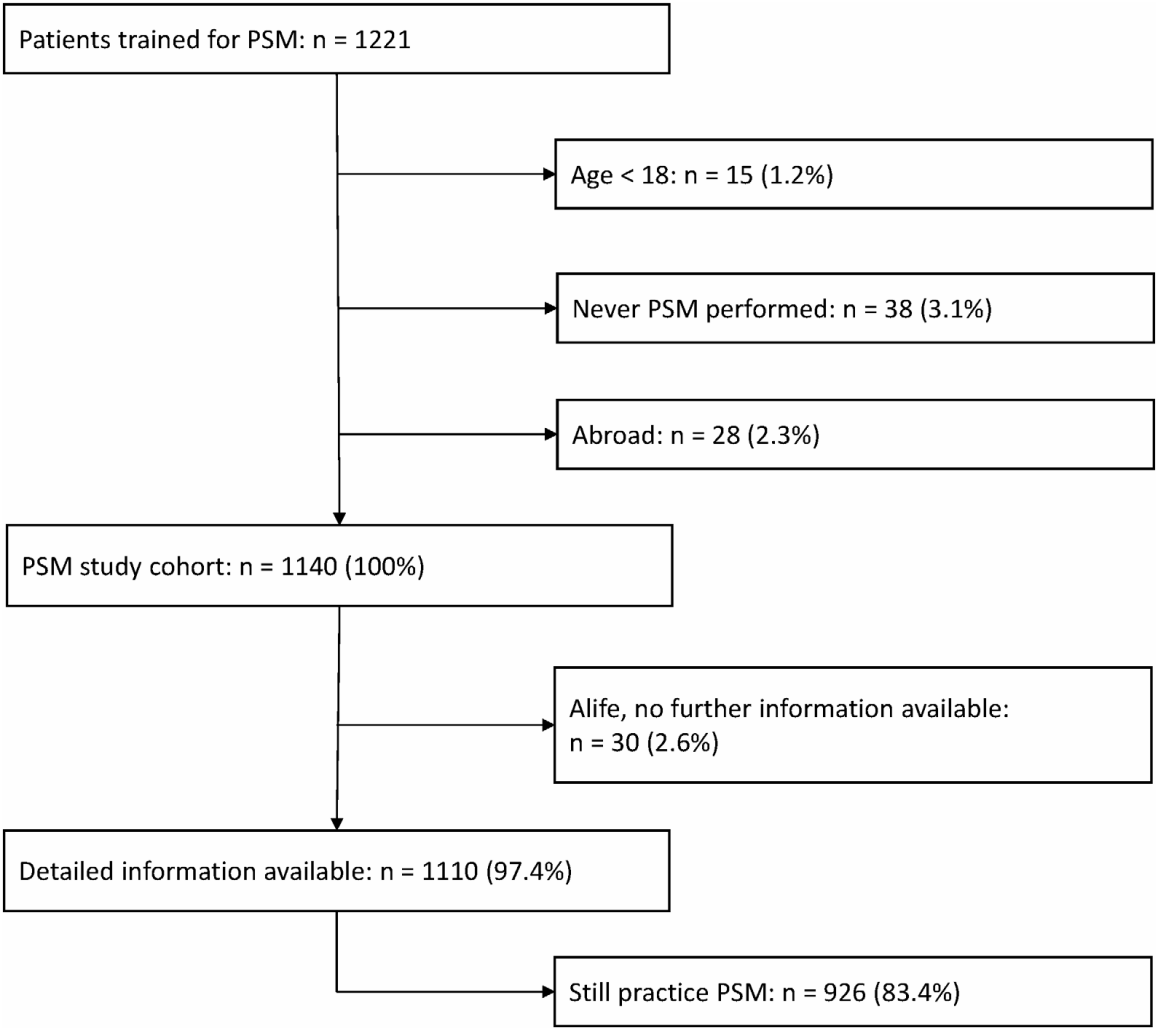
PSM study cohort.

Between 1998 and 2009, 1221 patients were trained for PSM (Fig. 1). Fifteen patients were excluded because of age<18 years (1.2%), 38 patients never performed PSM (3.1%), and 28 patients moved abroad (2.3%). The remaining 1140 patients constituted the PSM study cohort, representing 5818.9 patient-years. Survival status was available for all patients. Detailed information on complications was available for 97.4% of the patients, representing 5636.5 patient-years. Median follow-up was 4.3 years (range 0.2–12.8 years). Baseline characteristics of the study cohort and indications for oral anticoagulation are displayed in Table 1. Median age at the time of PSM training was 54.2 years (range 18.2–85.2) and 34.6% were women. Phenprocoumon was used as VKA almost exclusively (95.3%), and acenocoumarol or warfarin in the remaining cases.

**Table 1:** Baseline characteristics of the study cohort.

	Patients	Age	Female sex	Observation period	
	n (%)	median (range)	n (%)	patient-years	median (range)
**Overall**	1140 (100)	54.2 (18.2–85.2)	394 (34.6)	5818.9	4.3 (0.2–12.8)
**Venous thromboembolism**	463 (40.6)	48.5 (19.0–82.4)	199 (43.0)	2315.9	4.0 (0.2–12.7)
**Prosthetic heart valve**	365 (32.0)	55.0 (18.7–82.5)	104 (28.5)	2040.1	5.3 (0.8–12.8)
**Atrial fibrillation**	203 (17.8)	64.8 (18.2–85.2)	53 (26.1)	926.4	3.8 (0.2–12.6)
**Arterial thromboembolism**	54 (4.7)	57.3 (21.3–83.3)	19 (35.2)	319.8	5.5 (1.0–12.7)
**Others**	55 (4.8)	47.6 (20.9–82.2)	19 (34.5)	216.7	3.3 (0.4–11.3)

### Mortality

**Figure 2:**
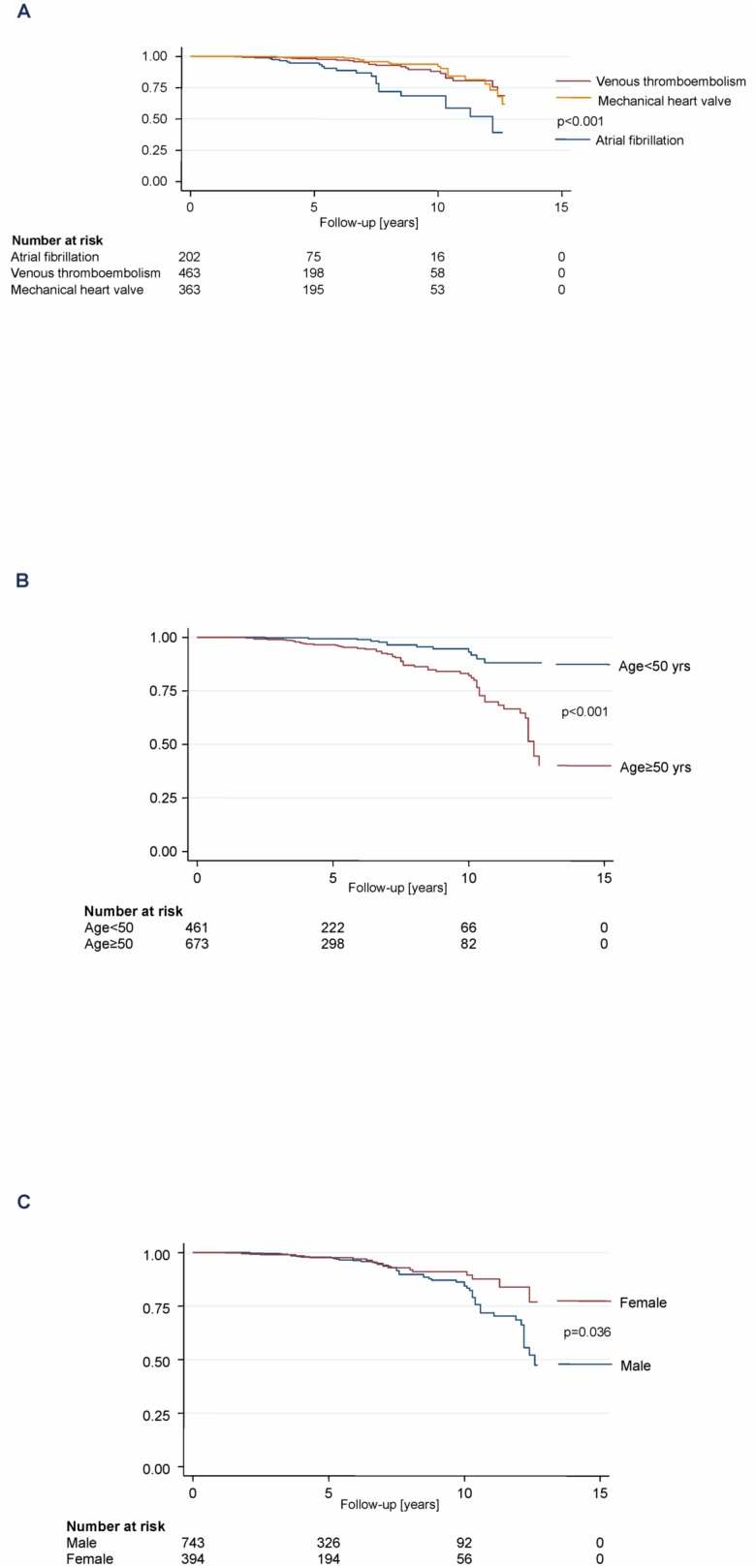
Survival estimates with regard to indications of oral anticoagulation, age, and sex.

Eighty patients died during the study period, constituting 1.4 deaths per 100 patient-years (Table 2). Kaplan-Meier survival estimates with regard to relevant subgroups are shown in Fig. 2. Mortality was higher among patients with atrial fibrillation (2.5 per 100 patient-years) compared with venous thromboembolism (1.1 per 100 patient-years) and prosthetic heart valve (1.0 per 100 patient-years; p<0.001). Mortality was higher in patients above 50 years (2.0 vs. 0.5 per 100 patient-years; p<0.001) and higher in men (1.6 vs. 1.0 per 100 patient-years; p = 0.036). However, when adjusting for differences in age between female and male using Cox regression, this effect was reduced and became non-significant. (Hazard Ratio (95% CI) 1.33 (0.79 to 2.24); p-value = 0.277).

**Table 2:** Mortality of PSM study cohort.

		All cause mortality	Event-related mortality	Not event-related mortality	Event-related + unknown cause
		n; deaths per 100 patient-years (95% CI)			
	**Overall** *n = 1140*	80; **1.4** (1.1–1.7)	5; **0.1** (0.02–0.2)	43; **0.7** (0.5–1.0)	37; **0.6** (0.4–0.9)
**Indication**	**Venous thromboembolism** *n = 463*	25; **1.1** (0.7–1.6)	2; **0.1** (0.01–0.3)	14; **0.6** (0.3–1.0)	11; **0.5** (0.2–0.8)
	**Prosthetic heart valve** *n = 365*	21; **1.0** (0.6–1.6)	1; **0.1** (0.001–0.3)	13; **0.6** (0.3–1.1)	8; **0.4** (0.2–0.8)
	**Atrial fibrillation** *n = 203*	23; **2.5** (1.6–3.7)	2; **0.2** (0.03–0.8)	10; **1.1** (0.5–2.0)	13; **1.4** (0.7–2.4)
	**Arterial thromboembolism** *n = 54*	4; **1.3** (0.3–3.2)	0; **0.0** (0.0–1.1)	3; **0.9** (0.2–2.7)	1; **0.3** (0.01–1.7)
	**Others** *n = 55*	7; **3.2** (1.3–6.7)	0; **0.0** (0.0–1.7)	3; **1.4** (0.3–4.0)	4; **1.8** (0.5–4.7)
**Age**	**<50 years** *n = 464*	13; **0.5** (0.3–0.9)	0; **0.0** (0.0–0.2)	10; **0.4** (0.2–0.7)	3; **0.1** (0.03–0.4)
	**≥50 years** *n = 676*	67; **2.0** (1.6–2.6)	5; **0.1** (0.04–0.3)	33; **0.9** (0.6–1.3)	34; **0.9** (0.6–1.3)
**Sex**	**Female** *n = 394*	21; **1.0** (0.6–1.5)	2; **0.1** (0.01–0.3)	14; **0.7** (0.4–1.1)	19; **0.9** (0.5–1.4)
	**Male** *n = 746*	59; **1.6** (1.2–2.1)	3; **0.1** (0.02–0.2)	29; **0.8** (0.5–1.1)	30; **0.8** (0.5–1.2)

CI, confidence interval.

### Thromboembolic Complications

**Figure 3:**
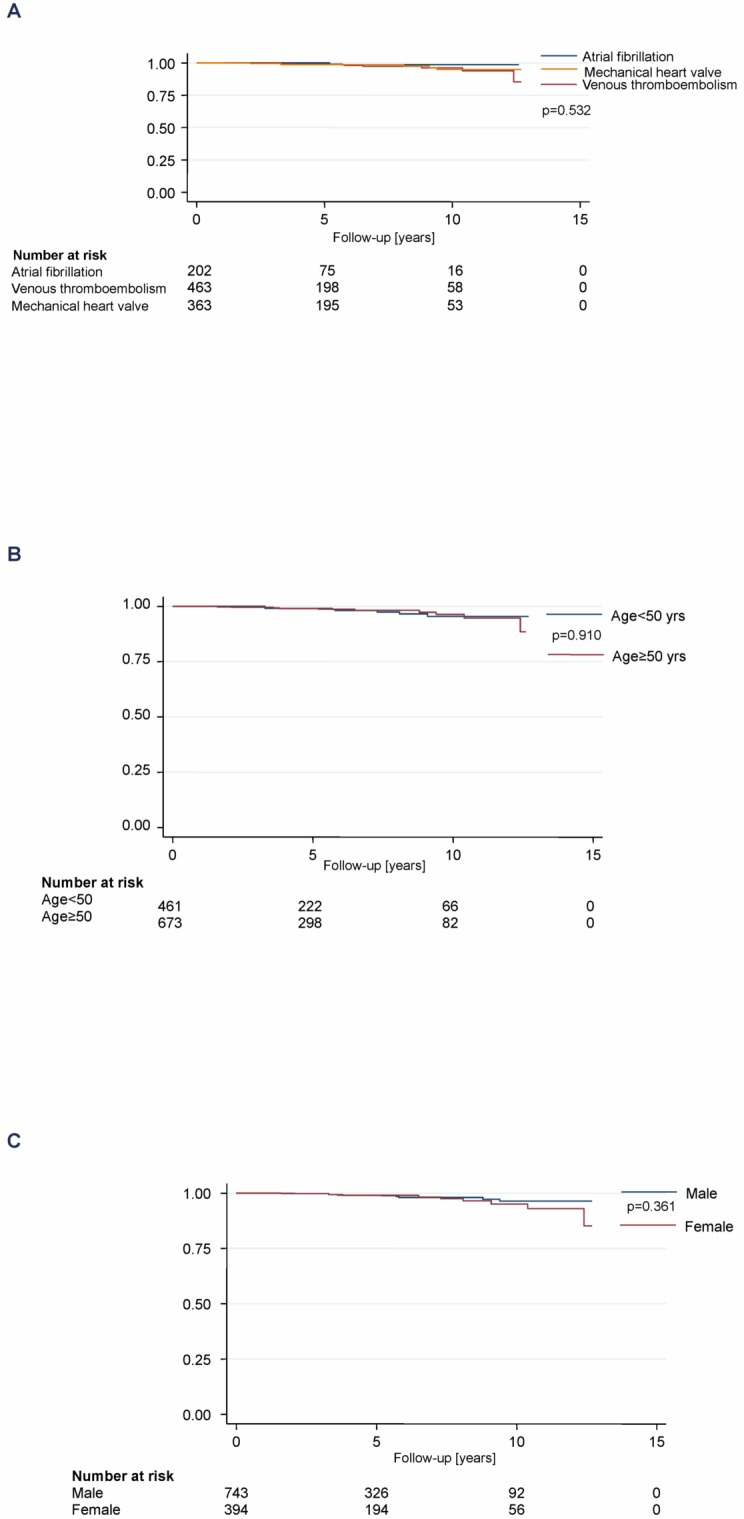
Thromboembolic complications by indications of oral anticoagulation, age, and sex.

Twenty-two venous and arterial thromboembolic complications were documented during the observation period, leading to a rate of 0.4 per 100 patient-years (95% CI 0.2–0.6; see Table 3). If unclear deaths were counted as thromboembolic complication, the rate would be 0.9 per 100 patient-years (95% CI 0.7–1.2). No differences could be found between the different subgroups (see Fig. 3).

**Table 3:** Thromboembolic and bleeding events.

		Thromboembolic events	Thromboembolic events + unclear deaths	Major bleeding events	Intracranial bleeding events
		numbers per 100 patient-years (95% CI)			
	**Overall** *n = 1110*	0.4 (0.2–0.6)	0.9 (0.7–1.2)	1.1 (0.9–1.5)	0.2 (0.1–0.3)
**Indication**	**Venous thromboembolism** *n = 451*	0.5 (0.2–0.9)	0.5 (0.3–0.9)	0.9 (0.6–1.4)	0.1 (0.01–0.3)
	**Prosthetic heart valve** *n = 356*	0.4 (0.2–0.8)	0.7 (0.4–1.2)	1.5 (1.0–2.2)	0.3 (0.1–0.7)
	**Atrial fibrillation** *n = 198*	0.1 (0.002–0.6)	1.3 (0.7–2.3)	1.1 (0.5–2.0)	0.1 (0.003–0.6)
	**Arterial thromboembolism** *n = 51*	0.3 (0.01–1.8)	0.6 (0.1–2.3)	1.7 (0.5–3.9)	0 (0–1.2)
	**Others** *n = 54*	0 (0–1.7)	1.8 (0.5–4.7)	0 (0–1.7)	0 (0–1.7)
**Age**	**<50 years** *n = 451*	0.4 (0.2–0.7)	0.5 (0.3–0.9)	0.7 (0.4–1.1)	0.04 (0.0–0.2)
	**≥50 years** *n = 659*	0.4 (0.2–0.7)	1.3 (0.9–1.8)	1.5 (1.1–2.0)	0.2 (0.1–0.5)
**Sex**	**Female** *n = 382*	0.5 (0.2–0.9)	0.7 (0.4–1.2)	1.3 (0.9–1.9)	0.2 (0.1–0.5)
	**Male** *n = 728*	0.3 (0.2–0.6)	1.1 (0.8–1.5)	1.1 (0.8–1.5)	0.1 (0.05–0.3)

CI, confidence interval.

### Major Bleedings

**Figure 4:**
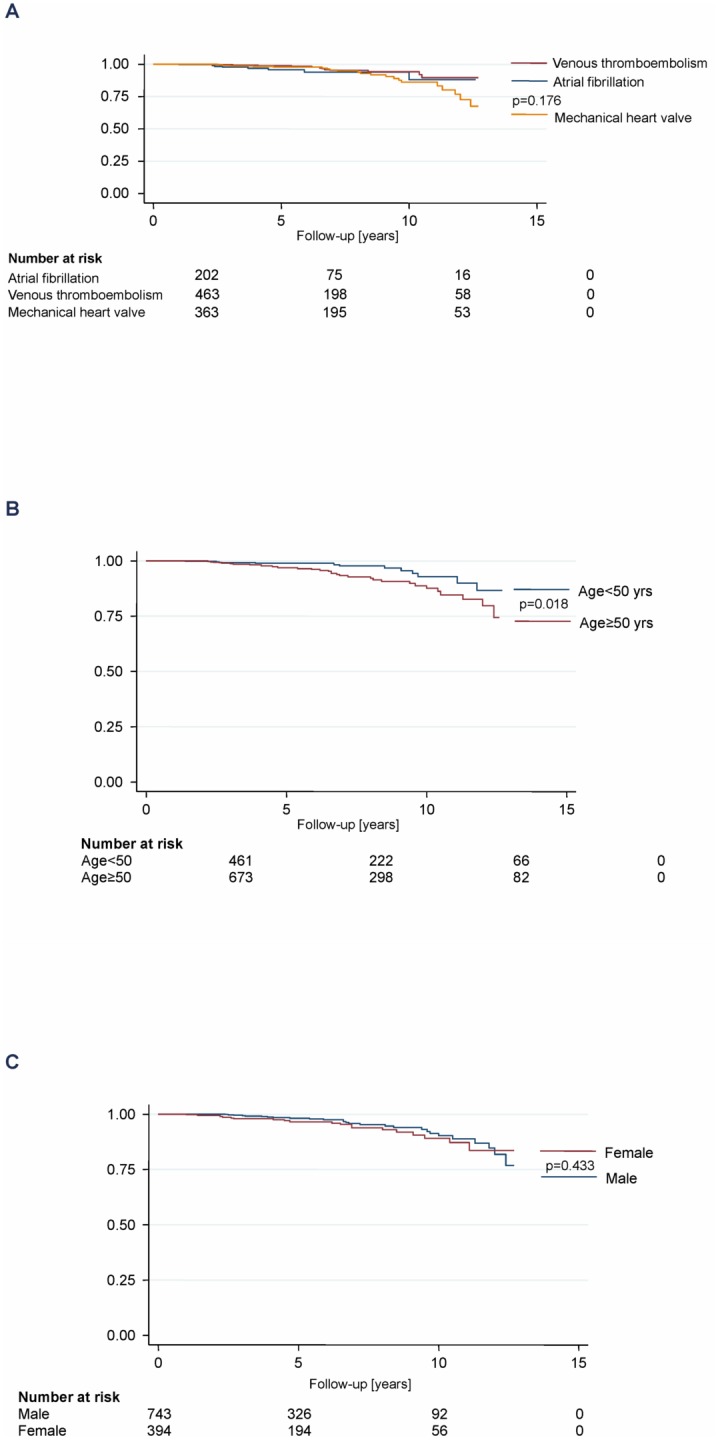
Major bleedings by indications of oral anticoagulation, age, and sex.

Sixty-six major bleedings were reported during the study period, corresponding to a rate of 1.1 per 100 patient-years (95% CI 0.9–1.5; Table 3). The rate was higher among patients older 50 years (1.5 per 100 patient-years; 95% CI 1.1–2.0) than in younger patients (0.7; 95% CI 0.4–1.1; Fig. 4). No differences were found between the established indications and between male and female patients. Nine intracranial bleedings were documented (0.2 per 100 patient-years; 95% CI 0.1–0.3) with a trend towards a higher rate in patients older 50 years (0.2 vs. 0.04 per 100 patient-years; 95% CI 0.1–0.5 vs. 0.0–0.2).

### Quality of Anticoagulation

INR data of 653 patients were available for analysis, corresponding to an observation period of 1743 patient-years (74,665 data points; 59% of the patients). Median observation period was 2.19 years (interquartile range [IQR] 0.98–3.97). The median time within the intended therapeutic range was 80% (IQR 66–89%). Median time in a safety range of 2.0 to 4.5 was 96% (IQR 89–99).

### Comparison with VKA Standard Care, and New Oral Anticoagulants

**Figure 5:**
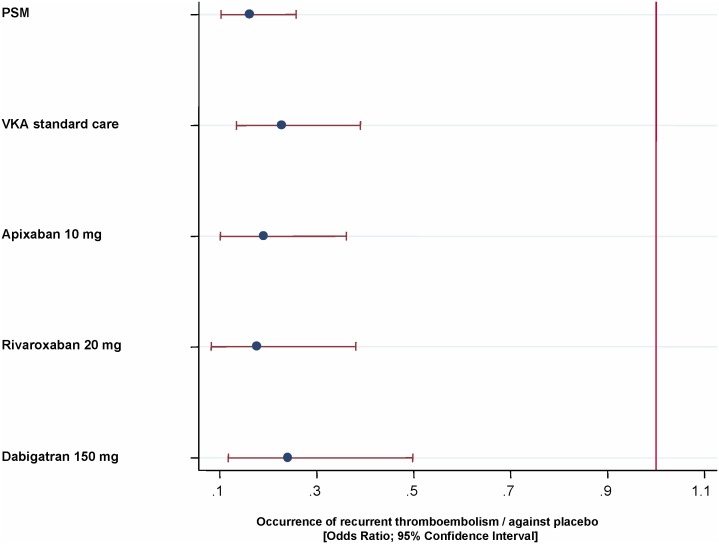
Efficacy of PSM in comparison to VKA standard care and new oral anticoagulants.

Occurrence of recurrent thromboembolism, as observed in a network meta-analysis, was at least comparable to mentioned treatments (Fig. 5). OR of PSM against placebo was 0.16 (0.10–0.26; p<0.001), for warfarin standard care 0.23(0.13–0.39; p<0.001), for rivaroxaban 0.18 (0.08–0.38; p<0.001), for dabigatran 0.24 (0.12–0.50; p<0.001) and for apixaban, 0.19 (0.10–0.36; p<0.001).

## Discussion

### Key Findings

Our results indicate that PSM of properly trained patients is safe and effective in a long-term real-life setting and robust across different clinical subgroups. Mortality is low, both overall and with regard to different indications of anticoagulation, sex and age. Thromboembolic events are very uncommon and the rate of major bleedings as well as intracranial bleedings is moderate. Furthermore, quality of anticoagulation therapy is high. Efficacy is comparable to VKA standard care and new oral anticoagulants.

### Comparison with other Studies

Randomized controlled trials, systematic reviews and individual patient data meta-analyses [4]–[6] consistently showed that PSM with VKA is effective. Nevertheless, many authors doubted that these results can be transferred to clinical practice straightforwardly because the experimental set-up within trials does not reflect daily routine [4], [5], [7]–[10], [14], [16] and study groups are prone being heavily selected [21], [22]. Moreover they criticised that follow-up periods within randomised studies are too short to extrapolate long-term consequences of PSM and called for population-based cohort studies to address these important points.

We are aware of two smaller observational studies investigating the efficacy and safety in patients with PSM. A retrospective analysis of 160 patients on PSM after implantation of a mechanical heart valve revealed a reduction in mortality and severe adverse events if compared with 260 patients on usual care (mean follow-up 8.6±2.1 years) [38]. In another investigation, 116 PSM patients out of 178 patients of a randomised controlled trial were re-analysed after 5 years [39]. Major bleedings were observed with a rate of 0.6 per 100 patient-years as well as 1.1 thromboembolic events per 100 patient-years. However, no mortality data and only few details of the study cohort were provided. Our study expands on their work reporting long-term efficacy and safety of PSM in real-life clinical practice, in a large prospective cohort and with regard to relevant outcome parameters and salient clinical subgroups.

### Strengths and Limitations

The strength of our investigation is the design of a prospective cohort study of all patients trained for PSM. The nationwide initiative “coagulationcare” covers about 90% of all trained patients in Switzerland and not one single patient was lost to follow-up. This makes any selection bias at the study level unlikely. Our study has several limitations. First, we cannot fully exclude that motivational or other factors may have influenced the referring decision of physicians and thus lead to a selected study group (selection at referral level). However, we believe that a systematic selection – if any – is unlikely. Second, we included patients from one country only, trained by one training program, using mostly phenprocoumon as anticoagulant. Although the training program was established according to current guidelines [18], [19], our results may need confirmation from other countries, using other training programs and anticoagulants such as warfarin. Finally, a direct comparison of death and survival rates with the general population on one hand and with other treatment schemes on the other hand was impossible. To overcome this limitation, we performed a network meta-analysis including PSM as well as VKA standard care and new oral anticoagulants. Furthermore, we put the results of our investigation into context with the results of large thromboembolism trials for different indications (Table S1).

### Who should Perform PSM?

In 2006, Douketis and Singh identified four requirements that patients qualifying for anticoagulant self-monitoring should fulfill; patients should necessitate long-term treatment, have no vision impairment that precludes appropriate testing, should have the cognitive ability to receive training and should be willing to take an active role in their illness [40]. Down these lines, today, a selection strategy based on existing guidelines targets at motivated patients who represent an important socio-economic group. In this group adherence to therapy, patient satisfaction and quality of life is very high. PSM also represents a rational treatment option in settings with limited access to (para-) medical care and thus qualifies for a variety of healthcare settings. PSM also has the potential to overcome well-known barriers of anticoagulant treatment prescription surveyed in the general practitioner setting [41]–[44]. We therefore call for efficient implementation strategies promoting PSM in clinical practice.

### Is there a Role for PSM in the Era of New Oral Anticoagulants?

In search of drugs with a more predictable effect lacking the need for routine monitoring, new, direct factor Xa and thrombin inhibitors have become available. A recent systematic review concluded that these drugs are a viable option, but benefits compared to warfarin are small and depend on the quality of anticoagulation, that is achieved with warfarin [45]. Ultimately, whether or not these new drugs are worth their money depends on the outcome of randomized controlled trials comparing PSM with new oral anticoagulants and cost effectiveness analyses considering changing costs and reimbursements. To date, efficacy and safety of new oral anticoagulants in real-life practice is largely unknown. Benefits may be reduced due to a lack of antidote, impaired adherence, and application to patients that would be excluded in clinical trials. Furthermore, new anticoagulants are not applicable in patients with mechanical heart valves and a relevant renal impairment [46].

### What are Implications for Research?

Unlike in other chronic clinical conditions such as asthma, arthritis or diabetes, evidence on the usefulness of PSM in oral anticoagulation remains less well established. When developing the research agenda for patient self-management of oral anticoagulation now, several lessons from other experiences can be learned. In the case of chronic obstructive pulmonary disease for instance, a Cochrane review recently concluded that heterogeneity in interventions, study populations, follow-up time, and outcome measures impeded from drawing firm recommendations [3]. Another recent review examining the usefulness of self-measured blood pressure in hypertension concluded that long-term benefits remained uncertain [2]. Besides replications of our study in other countries and using other anticoagulants, we call for systematic investigations specifying those patient profiles benefitting most from PSM. Finally, cost effectiveness analyses considering changing costs and reimbursements in the context of new anticoagulants are needed.

In conclusion, PSM of properly trained patients is effective and safe in a real-life setting and robust across clinical subgroups. It represents a rational strategy to provide high quality anticoagulation therapy in many service settings including those with limited access to (para) medical care or rural areas, where patients need travelling greater distances for healthcare and thus deserves being broadly promoted.

## Supporting Information

Table S1 Complications of PSM in a long-term real-life setting in contrast to major anticoagulation studies.
(DOCX)

Table S2 PSM training package.
(DOCX)
