# Supplementary material for: Patient Self-Management of Oral Anticoagulation with Vitamin K Antagonists in Everyday Practice: Efficacy and Safety in a Nationwide Long-Term Prospective Cohort Study
Source: PLoS One. 2014 Apr 18;9(4):e95761. doi: 10.1371/journal.pone.0095761 (PMC3991723; doi:10.1371/journal.pone.0095761)
Supplement: Table S1 — Complications of PSM in a long-term real-life setting in contrast to major anticoagulation studies. (DOCX) [file pone.0095761.s001.docx]

| **Author** | **Year** | **Patients** | **Design** | **Treatment** | **Indication** | **Age** | **Observation period**^&&^ | **Mortality*** | **TE events*** | **Major bleedings*** | **Time in target range** |
| --- | --- | --- | --- | --- | --- | --- | --- | --- | --- | --- | --- |
| Nagler M et al | 2013 | 1221 | Cohort | PSM | AF^°°^, VTE^++^, MHV^**^, | 53.7° | 51.6° | 1.4 | 1.0^#^ | 1.2 | 80.0%° |
| Matchar DB et al[[1](#_ENREF_1)] | 2010 | 1463 / 1452 | RCT | PST^##^ vs. AC^§^ | AF, MHV | 66.6^+^ / 67.4^+^ | 36^+^ | 3.4 / 3.7 | 2.2 / 2.4 | 4.0 / 4.7 | 66.2% |
| Patel MR et al[[2](#_ENREF_2)] (ROCKET AF) | 2011 | 7131 / 7133 | RCT | Rivaroxaban vs. Warfarin usual care | AF | 73^+^ / 73^+^ | 19.7° | 1.9 / 2.2 | 3.1^§§^ / 3.6^§§^ | 3.6 / 3.4 | 55%^+^ |
| Connolly SJ et al[[3](#_ENREF_3)] (RE-LY) | 2009 | 6015 / 6076 / 6022 | RCT | Dabigatran 110 mg vs. 150 mg vs. Warfarin usual care | AF | 71.4^+^ / 71.5^+^ / 71.6^+^ | 24.0° | 3.8 / 3.6 / 4.1 | 2.4^$^ / 2.0^$^ / 2.3^$^ | 2.7 / 3.1 / 3.4 | 64%° |
| Granger CB et al[[4](#_ENREF_4)] (ARISTOTLE) | 2011 | 9120 / 9081 | RCT | Apixaban vs. Warfarin usual care | AF | 70° / 70° | 21.6° | 3.5 / 3.9 | 1.8^$$^ / 2.3^$$^ | 2.1 / 3.1 | 66%° / 62%^+^ |
| Cannegieter SC et al[[5](#_ENREF_5)] | 1995 | 1608 | Cohort | VKA | MHV | 50 – 69 (62% of the patients) | 48^+^ | 3.9 | 0.7*** | 2.7 | 61% |
| Koertke H et al[[6](#_ENREF_6)] | 2007 | 2673 | RCT | Low dose vs. conventional dose PSM | MHV | 59.2 / 60.0 | 24 | 2.9 | 0.5^&^ | 2.4^&^ | 77% / 75% |
| Ridker PM et al[[7](#_ENREF_7)] (PREVENT) | 2003 | 253 / 253 | RCT | Placebo vs. Warfarin^$$$^ | Recurrent VTE | 53 / 53 | 25.2^+^ | 1.4 / 0.7 | 7.2^+++^ / 2.6^+++^ | 0.4 / 0.9 | n.s. |

* per 100 patient-years; ^#^ thromboembolic events and unclear death; ° median; ^+^mean; ^§^ anticoagulation clinic; ++ venous thromboembolism; ** mechanical heart valve; °° atrial fibrillation; ^##^ patient self-testing; ^§§^ stroke or systemic embolism only; ^$^ stroke, systemic embolism, myocardial infarction and pulmonary embolism; ^$$^ stroke, systemic embolism, myocardial infarction and pulmonary embolism; ***cerebral infarction, peripheral embolism and valve thrombosis only; ^+++^ venous thromboembolism only; ^$$$^ anticoagulation clinic?; ^&^ only major complications, that required hospitalization; ^&&^ month

1. Matchar DB, Jacobson A, Dolor R, et al. Effect of home testing of international normalized ratio on clinical events. N Engl J Med 2010;**363**(17):1608-20 doi: 10.1056/NEJMoa1002617[published Online First: Epub Date]|.

2. Patel MR, Mahaffey KW, Garg J, et al. Rivaroxaban versus warfarin in nonvalvular atrial fibrillation. N Engl J Med 2011;**365**(10):883-91 doi: 10.1056/NEJMoa1009638[published Online First: Epub Date]|.

3. Connolly SJ, Ezekowitz MD, Yusuf S, et al. Dabigatran versus warfarin in patients with atrial fibrillation. N Engl J Med 2009;**361**(12):1139-51 doi: 10.1056/NEJMoa0905561[published Online First: Epub Date]|.

4. Granger CB, Alexander JH, McMurray JJ, et al. Apixaban versus warfarin in patients with atrial fibrillation. N Engl J Med 2011;**365**(11):981-92 doi: 10.1056/NEJMoa1107039[published Online First: Epub Date]|.

5. Cannegieter SC, Rosendaal FR, Wintzen AR, et al. Optimal oral anticoagulant therapy in patients with mechanical heart valves. The New England journal of medicine 1995;**333**(1):11-7 doi: 10.1056/NEJM199507063330103[published Online First: Epub Date]|.

6. Koertke H, Zittermann A, Tenderich G, et al. Low-dose oral anticoagulation in patients with mechanical heart valve prostheses: final report from the early self-management anticoagulation trial II. Eur Heart J 2007;**28**(20):2479-84 doi: 10.1093/eurheartj/ehm391[published Online First: Epub Date]|.

7. Ridker PM, Goldhaber SZ, Danielson E, et al. Long-term, low-intensity warfarin therapy for the prevention of recurrent venous thromboembolism. N Engl J Med 2003;**348**(15):1425-34 doi: 10.1056/NEJMoa035029[published Online First: Epub Date]|.
