# Supplementary material for: Patient Self-Management of Oral Anticoagulation with Vitamin K Antagonists in Everyday Practice: Efficacy and Safety in a Nationwide Long-Term Prospective Cohort Study
Source: PLoS One. 2014 Apr 18;9(4):e95761. doi: 10.1371/journal.pone.0095761 (PMC3991723; doi:10.1371/journal.pone.0095761)
Supplement: Table S2 — PSM training package. (DOCX) [file pone.0095761.s002.docx]

| **Phase of training** | **Time frame** | **Type of training** | **Supervision** | **Contents** |
| --- | --- | --- | --- | --- |
| Training course | Full day | Teaching lessons  Workshops  Supervised testing  Self-testing  q/a session | Specialised physician  Specialised paramedical staff | Principles of coagulation and anticoagulation  Drugs that influence haemostasis  Determination and interpretation of INR values  Handling of the coagulometer  Dose adjustments  Procedure in case of high respective low INR values  Bleeding complications  Side effects  Interaction with drugs and alimentation  Procedure in case of operations, dental procedures, vaccination and accidents  Bridging formula  Important resources for help |
| Self training period | Four to eight weeks | Self-testing | 24-hour hotline  Online material  e-mail contact  Family physician | Handling of the coagulometer  Dose adjustments  Control of the coagulometer by parallel measurements with the family physician |
| Follow up visit | One hour visit | Supervised testing | Specialised physician | Repetition of the most important concepts  Control of the coagulometer with standard laboratory method  Dose adjustments |
| Long-term support | Long-term | Feedback by request | 24-hour hotline  Online material  e-mail contact  Family physician | Control of the coagulometer by parallel measurements with the family physicians (1-2 times yearly)  Dose adjustments  All issues |
